# Clinical features, genomic profiling, and outcomes of adult patients with unifocal Langerhans cell histiocytosis

**DOI:** 10.1186/s13023-023-02989-8

**Published:** 2023-11-30

**Authors:** Min Lang, Hua-cong Cai, He Lin, Long Chang, Jia-wen Dai, Jia Chen, Ming-hui Duan, Dao-bin Zhou, Gaurav Goyal, Xin-xin Cao

**Affiliations:** 1 Department of Hematology, Peking Union Medical College Hospital, Chinese Academy of Medical Sciences and Peking Union Medical College, Beijing, 100730 China; 2 State Key Laboratory of Complex Severe and Rare Diseases, Peking Union Medical College Hospital, Chinese Academy of Medical Sciences and Peking Union Medical College, Beijing, China; 3 Division of Hematology-Oncology, University of Alabama at Birmingham, 1802 6th Ave S, NP 2500, Birmingham, AL 35294 USA

**Keywords:** Langerhans cell histiocytosis, Unifocal, Target gene sequencing, Prognosis

## Abstract

**Background:**

Langerhans cell histiocytosis (LCH) is a rare highly heterogeneous histiocytosis, which can be divided into single system and multiple system disease according to site of involvement. There is a paucity of studies examining unifocal LCH in adults in the molecular era.

**Results:**

We retrospectively analysed records from 70 patients with unifocal LCH. The median age at diagnosis was 36 years (18–69). The most common organ involved was the bone (70.0%), followed by pituitary gland (7.1%). Target gene sequencing of lesion tissues was performed on 32 of the 70 patients. *MAPK/PI3K* pathway alterations were observed in 78.1% of the patients; the most common mutations included *BRAF*^*V600E*^ (28.1%), *MAP2K1* (18.8%) and *PIK3CA* (9.4%). After a median follow-up time of 39.4 months (0.7–211.8), 10 (14.3%) patients developed disease progression, of whom 4 had local recurrence, 2 progressed to single-system multifocal and 4 progressed to multiple system LCH. The 3-year progression-free survival (PFS) was 81.9%. Univariate analysis showed that age < 30 years at diagnosis was associated with worse 3-year PFS (52.2% vs. 97.0%, *p* = 0.005). The 3-year overall survival was 100%.

**Conclusions:**

In our large cohort of adults with unifocal LCH, we found that prognosis of unifocal LCH in adults was very good, and age < 30 years at diagnosis was associated with increased relapse risk.

**Supplementary Information:**

The online version contains supplementary material available at 10.1186/s13023-023-02989-8.

## Background

Langerhans cell histiocytosis (LCH) is a rare type of histiocytosis that has marked clinical heterogeneity [1]. The incidence rate for LCH in adult is approximately 1–2 cases per million [2]. LCH can be divided into unifocal, single-system pulmonary, single system multifocal (SS-M) and multisystem (MS) disease according to the site of involvement [3]. The involved organs mainly include the bone, pituitary gland, lung, skin, thyroid gland, lymph nodes, liver, spleen and bone marrow, among which the liver, spleen and bone marrow are considered the risk organs [4]. The *BRAF*^*V600E*^ mutation was first reported in 57% of LCH samples in 2010, which solidified the notion that LCH was a clonal neoplasm [5]. Since then, other gene mutations, including *MAP2K1* and *MAP3K1*, have been found as well [6]. Our previous study showed that although *BRAF* or *MAP2K1* alterations were present in nearly 90% of adults with LCH, unlike in pediatric patients, *BRAF*^*V600E*^ occurred in only 31.5% of adult LCH patients, while *BRAF*^*indel*^ was identified in 28.8% of patients [7]. These results indicate that the pathophysiology may be different between pediatric and adult LCH. Given the biologic differences, we cannot extrapolate data from pediatric studies. The definition of unifocal is a solitary lesion involving any organ [3]. There are currently very few studies examining single-system unifocal LCH in adults. One of the largest contemporary case studies included 44 patients and showed that the prognosis for this disease subtype was excellent [8]; however, the mutational status of the patients was not reported in that study. The objective of this single-centre retrospective study was to describe the clinical features, somatic alterations, treatments, and prognosis of adults with unifocal LCH.

## Methods

### Patients

This retrospective study included adult patients diagnosed with unifocal LCH at Peking Union Medical College Hospital (Beijing, China) from September 2001 to December 2022. We included patients ≥ 18 years old with biopsy proven unifocal LCH in this analysis. Isolated pulmonary LCH was excluded. The histological findings were consistent with LCH on the basis of the World Health Organization classification of hematopoietic neoplasms [9]. All patients underwent full body 18F-fluorodeoxyglucose positron emission tomography (FDG-PET) and/or full body computed tomography (CT) to exclude involvement of other organs. Full body FDG-PET and CT usually did not include the brain, but patients with pituitary involvement all have had cerebral MRI. Patients were regular followed up every 6 months. For asymptomatic patients, a total body CT examination was required at least once a year. The study was conducted in accordance with the ethical standards of the 1964 Declaration of Helsinki and its subsequent amendments. The study was approved by the Ethics Committee of Peking Union Medical College Hospital.

### Data collection

In this study, patient demographics, disease characteristics and treatments were collected through electronic medical records. Demographic information included the gender of the patient and age at diagnosis. Disease characteristics included affected organs, clinical manifestations, laboratory examination including complete blood count, liver function tests. Radiological data, including thoracic, abdominal and pelvic CT, cerebral magnetic resonance imaging (MRI), radionuclide bone scans or FDG-PET, were collected. Genetic testing via targeted sequencing of 183 genes (Additional file 1: Table S1) was performed when sufficient DNA could be extracted from formalin-fixed paraffin-embedded (FFPE) lesion biopsy samples, as previously described [7].

### Treatment

This study retrospectively collected the first-line treatment for all patients. The initial treatment included local or systemic therapy. Local therapy included surgery and radiation therapy, and systemic therapy included chemotherapy such as methotrexate combined with cytarabine (MA) [10], vindesine and prednisone (VP)-based regimens [11] and cytarabine monotherapy.

### Patient outcomes

Patient outcomes were collected by clinical follow-up and telephone follow-up, and the last follow-up was on December 31, 2022. If the patient was not contacted at the last follow-up, we analysed the patients' conditions between diagnosis and the last follow-up record. Progression-free survival (PFS) was defined as the duration from diagnosis of LCH to the date of disease progression or death from any cause or last follow-up for patients with no recorded date of progression or death, with censoring for cases that were lost to follow-up. Overall survival (OS) was defined as the duration from the diagnosis of LCH to the date of death from any cause.

### Statistical analysis

All statistical analyses were performed using SPSS 25.0 statistical software (SPSS Inc., Chicago, Illinois, USA). Descriptive statistics were used to summarize the demographic profile and disease characteristics of the patients. Fisher’s exact test was used to compare categorical variables, and the Mann‒Whitney test was used to compare continuous variables. PFS and OS were estimated based on Kaplan‒Meier method. Univariate analysis Cox regression model was used to estimate OS and PFS-related factors. *p* < 0.05 was considered statistically significant.

## Results

### Demographics and disease characteristics

**Fig. 1:**
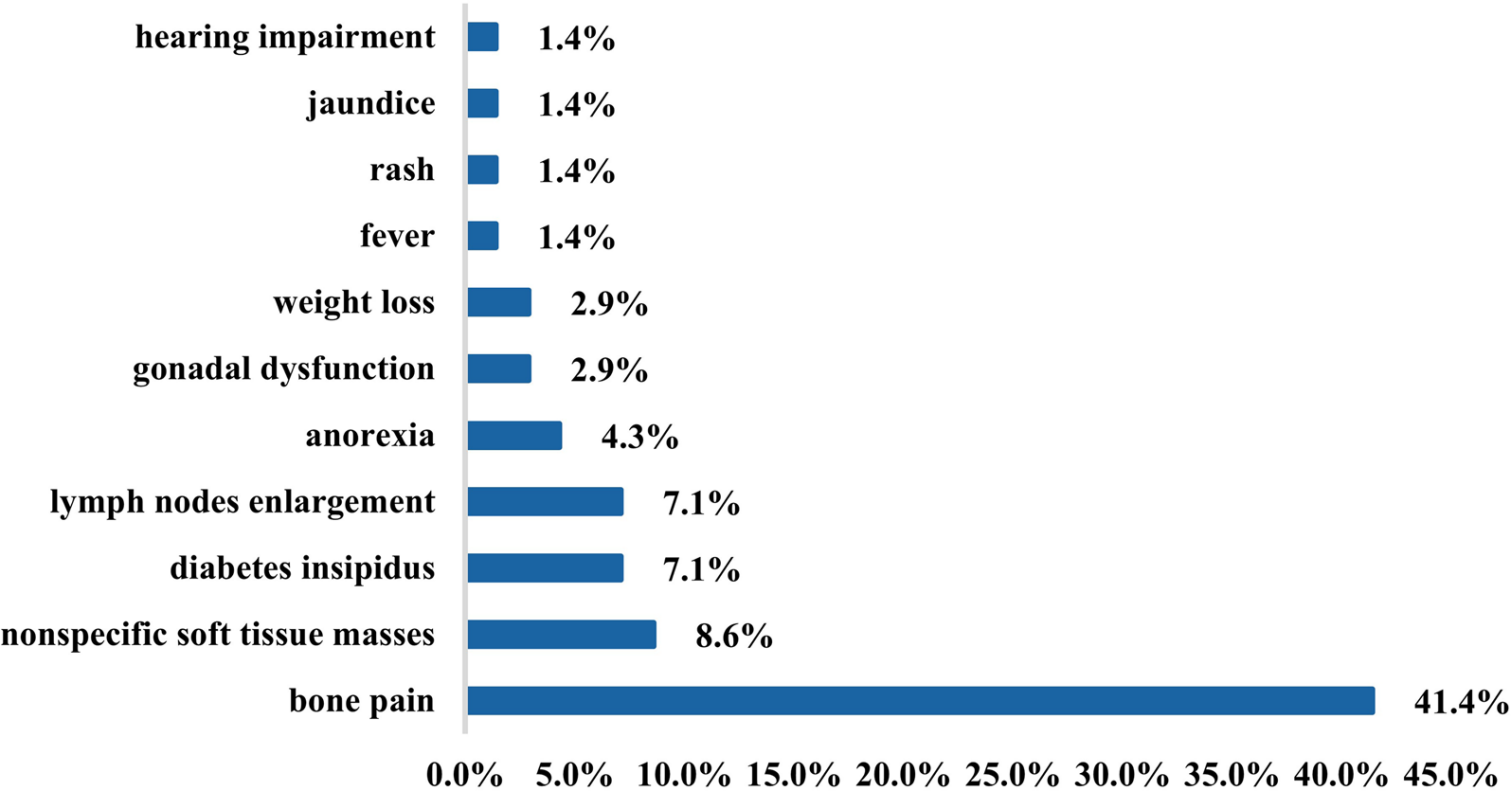
Percentage of clinical manifestations of adult patients with unifocal LCH

We included 70 patients in the study, including 42 males (60%) and 28 females (40%), with a 1.5:1 male to female ratio. The median age at diagnosis was 36 years (18–69), and the median duration from symptom onset to diagnosis was 2.0 months (0–50.0). The proportion of patients who underwent full body CT scans at diagnosis was 84.3% (n = 59), and FDG-PET was 51.4% (n = 36), and MRI brain was 27.1% (n = 19). All the patients underwent at least one full body radiological examination. Among the entire cohort, 68 patients (97.1%) had clinical manifestations leading to diagnosis (Fig. 1). The most common clinical manifestations were bone pain, accounting for 41.4% (n = 29), followed by nonspecific soft tissue masses 8.6% (n = 6), diabetes insipidus 7.1% (n = 5) and lymph node enlargement 7.1% (n = 5). Three patients had anorexia (4.3%). Gonadal dysfunction and weight loss were present in two (2.9%) patients each. Fever, rash, jaundice, and hearing impairment were found in one (1.4%) patient each. The most commonly affected organs were the bone, accounting for 70.0% (n = 49), followed by pituitary gland 7.1% (n = 5), lymph nodes 5.7% (n = 4), skin 4.3% (n = 3), liver 4.3% (n = 3) and thyroid 1.4% (n = 1). Other affected organs included eyelid, submandibular gland, parotid gland, digestive tract and epidural mass in one case each (Table 1). It is worth mentioning that we described three patients with unifocal liver involvement, and their liver enzymes were as follows: alanine aminotransferase (ALT) range 22–82 U/L, aspartate transaminase (AST) range 21–64 U/L, alkaline phosphatase (ALP) range 112–260 U/L, γ-glutamyl transpeptidase (GGT) range 75–335 U/L, total bilirubin and direct bilirubin were normal.

**Table 1:** Demographics and clinical characteristics of adult patients with unifocal LCH

Characteristic	
Sex	
Male, n (%)	42 (60.0)
Female, n (%)	28 (40.0)
Age at diagnosis, years, median (range)	36 (18–69)
Time from onset to diagnosis, months, median (range)	2.0 (0–50.0)
Clinical manifestations	
Yes, n (%)	68 (97.1)
No, n (%)	2 (2.9)
Organ involvement	
Bone, n (%)	49 (70.0)
Pituitary, n (%)	5 (7.1)
Lymph nodes, n (%)	4 (5.7)
Skin, n (%)	3 (4.3)
Liver, n (%)	3 (4.3)
Thyroid, n (%)	1 (1.4)
Eyelid, n (%)	1 (1.4)
Submandibular gland, n (%)	1 (1.4)
Parotid gland, n (%)	1 (1.4)
Digestive tract, n (%)	1 (1.4)
Epidural mass, n (%)	1 (1.4)

### Past and family medical history

Of the 70 patients, two had a history of previous cancer. One patient with skin involvement LCH had thyroid cancer that had been surgically removed before the diagnosis of LCH. Lung adenocarcinoma was found in one patient at the time of diagnosis of bone-involved LCH. Twenty-five patients (35.7%) had a smoking history, and the median smoking index was 8.1 pack-years (2.4–75.0). None of the patients had a family history of LCH or other histiocytic tumours.

### Genomic profiling

**Fig. 2:**
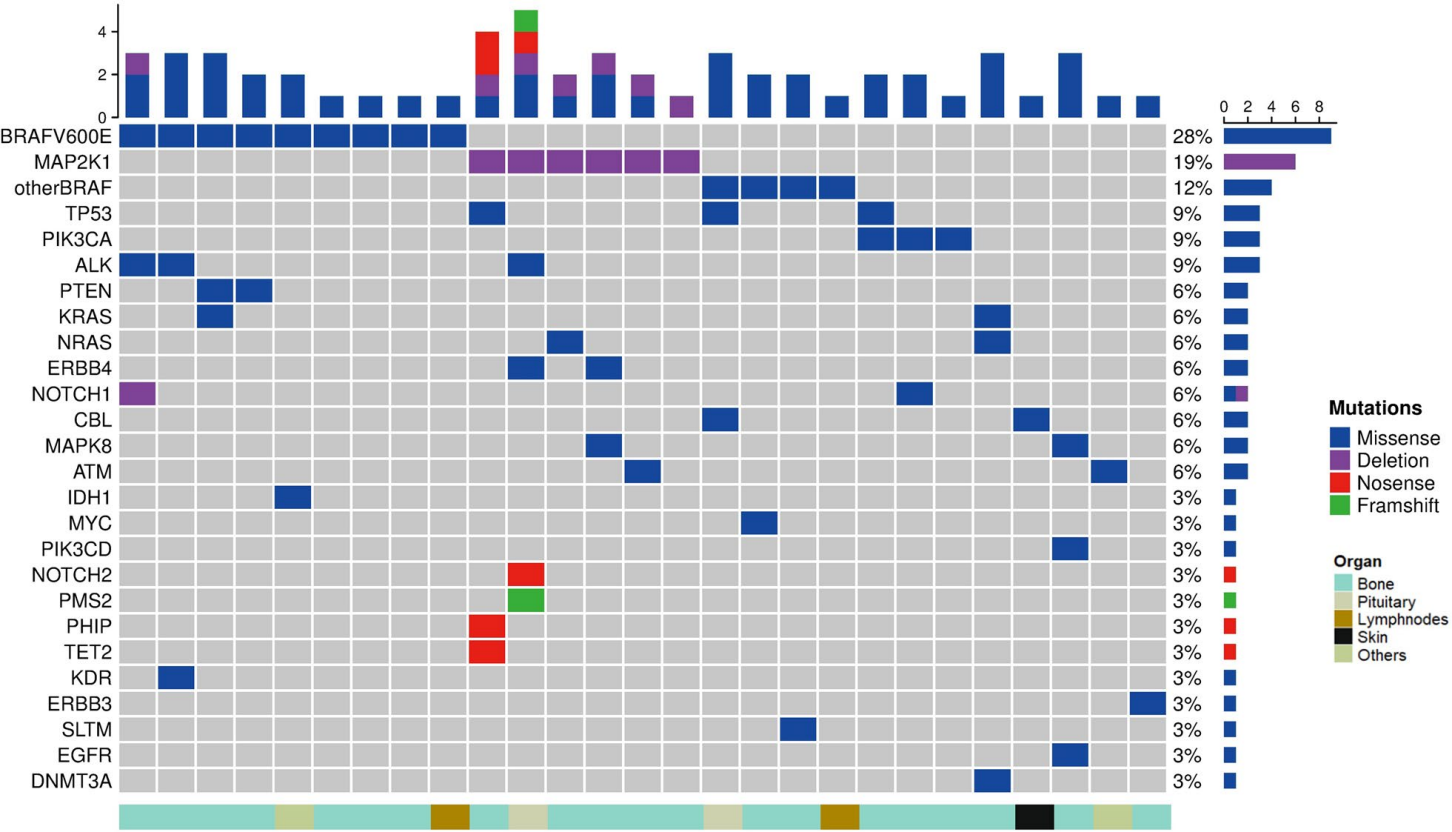
Target gene sequencing of lesion tissues of adult patients with unifocal LCH

Target gene sequencing of lesion tissues was performed on 32 of the 70 patients (Additional file 1: Table S2). Five patients had no mutations, including two bone, one lymph node, one skin and one liver involvement. *MAPK/PI3K* pathway alterations were present in 78.1% of the patients (n = 25). The frequencies of *BRAF*^*V600E*^ mutation, *MAP2K1* mutation and *PIK3CA* mutation were 28.1% (n = 9), 18.8% (n = 6) and 9.4% (n = 3), respectively. *KRAS* mutations were detected in two patients (6.3%), *NRAS* in two (6.3%), and *PIK3CD* in one (3.1%). None of the patients had the *BRAF*^*indel*^. In addition to the *BRAF*^*V600E*^ mutation, other *BRAF* mutations were also detected, including *BRAF*^*V600D*^, *BRAF*^*G466E*^, *BRAF*^*E501K*^ and *BRAF*^*T599I*^ in one patient each (3.1%) (Fig. 2). *BRAF*^*V600E*^ mutations and *MAP2K1* mutations were not associated with the type of organ involved.

### Treatments

**Fig. 3:**
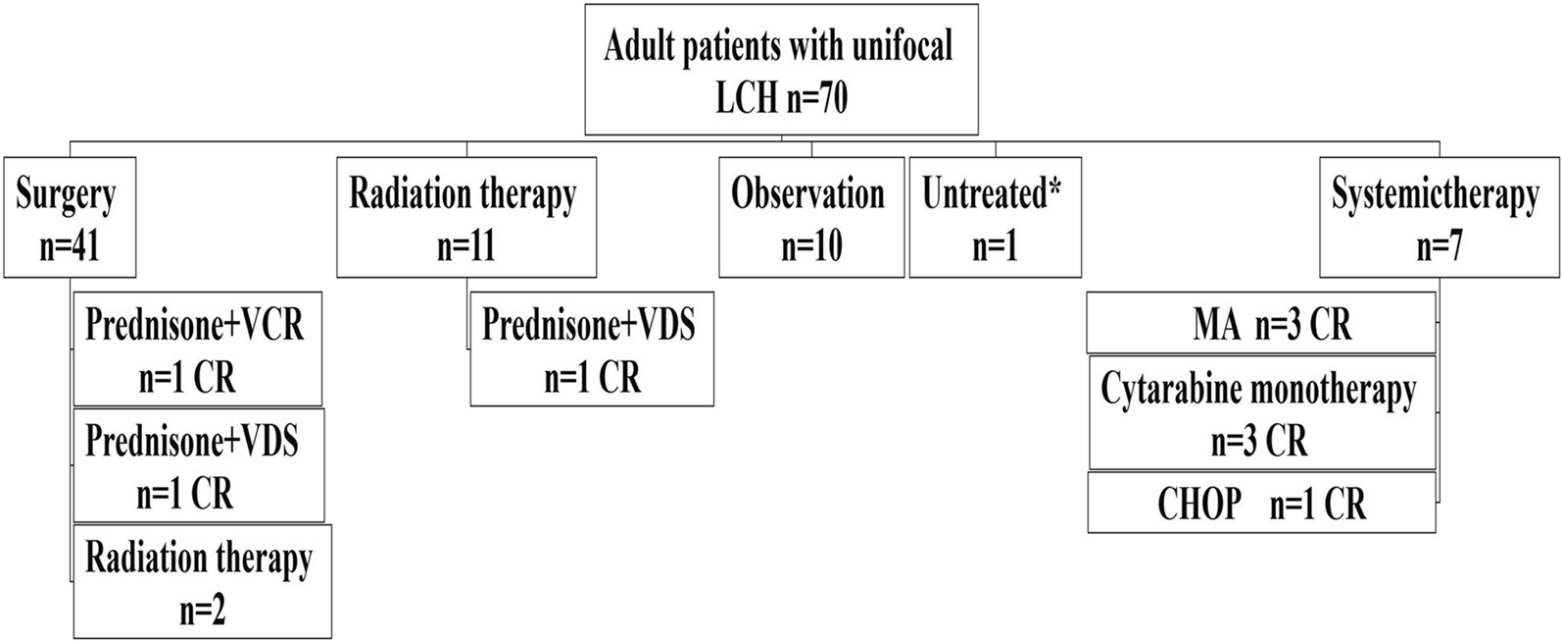
Treatments and outcomes of adult patients with unifocal LCH. *One patient had not yet begun treatment at the time of the last follow-up. *CR* complete response, *VCR* vincristine, *VDS* vindesine, *MA* methotrexate combined with cytarabine, *CHOP* cyclophosphamide, epirubicin, vindesine, prednisone

The initial treatment for the cohort is illustrated in a flow diagram in Fig. 3. One of the three patients with isolated liver involvement had not yet begun treatment at the time of last follow-up. Forty-one patients (58.6%) received surgical resection for first-line treatment. Because the surgical resection was not complete, two patients received postoperative radiation therapy, and two patients received postoperative VP-based chemotherapy. Eleven patients (15.7%) received radiation, and one patient received VP-based chemotherapy after radiotherapy to consolidate the treatment effect. Seven patients (10.0%) received systemic therapy, including MA for three patients, cytarabine monotherapy (100 mg/m^2^ subcutaneous injection, day1-5 every month, 12 months) for three patients, and VP-based therapy for one patient. Only 10 patients (14.3%) were observed. In addition to the patient who had not yet begun treatment, two of the three patients with liver involvement were treated with a cytarabine monotherapy regimen, and both achieved complete response.

### Prognosis

**Fig. 4:**
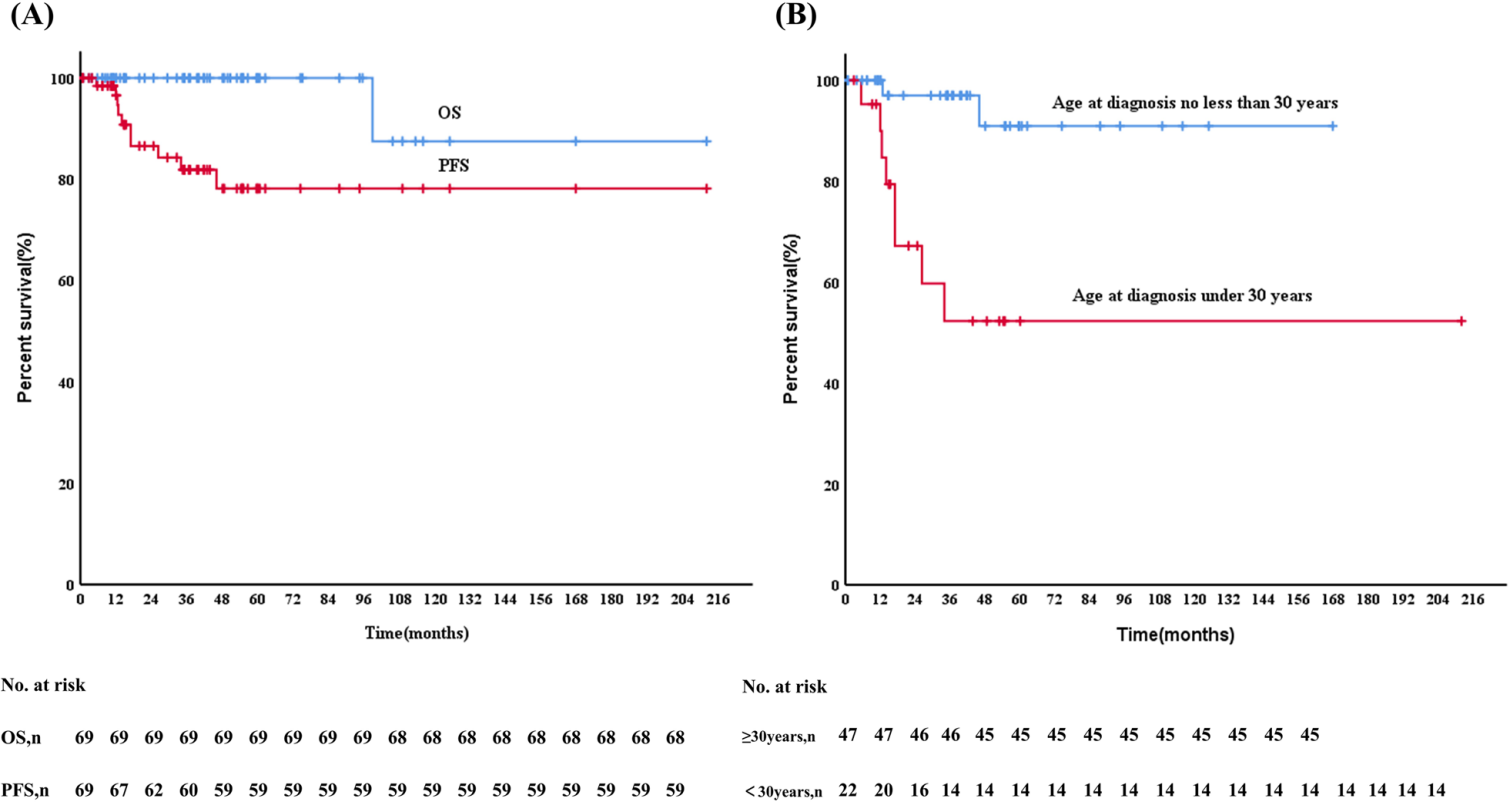
Progression-free survival (PFS) and overall survival (OS) of adult patients with unifocal LCH (**A**), and PFS (**B**) according to age at diagnosis

**Fig. 5:**
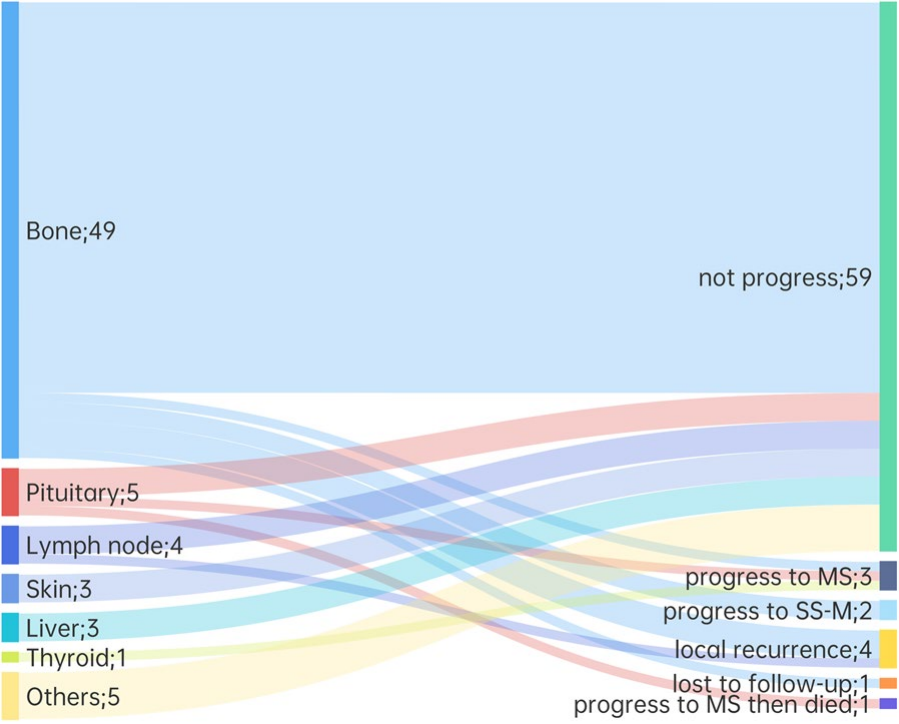
Progression pattern of each affected organ of adult patients with unifocal LCH. *MS* multiple system, *SS-M* single-system multifocal; Others: eyelid, submandibular gland, parotid gland, digestive tract and epidural mass in one case each

Of the 70 patients, one patient was lost to follow-up, and follow-up data were available for 69 patients. The median follow-up duration was 39.4 months (range 0.7–211.8 months). Ten (14.3%) patients developed disease progression, of whom 4 had local recurrence, 2 progressed to SS-M and 4 progressed to MS LCH. The 3-year PFS was 81.9% for all patients (Fig. 4A). The progression rate of bone involvement was 12.2% (6/49), and it was 40.0% (2/5) for pituitary involvement, and 25.0% (1/4) for lymph node involvement. Among the 6 patients with bone involvement who had disease reactivation, 3 patients had local recurrence, 2 progressed to SS-M and 1 to MS. The affected site of the patient who progressed to MS was in the mandible. In addition, two patients with pituitary involvement and one patient with thyroid involvement progressed to MS, and one patient with lymph node involvement had local recurrence (Fig. 5). Factor associated with worse 3-year PFS was age < 30 years at diagnosis (52.2% vs. 97.0%, *p* = 0.005) (Fig. 4B). During follow-up, one patient with initial pituitary involvement progressed to MS after 34 months. This patient died at 98.8 months following the initial LCH diagnosis, due to an infection after chemotherapy for LCH progression. The 3-year OS rate was 100% (Fig. 4A). Organ involvement, treatment option, *BRAF*^*V600E*^ mutations and *MAP2K1* mutations were not associated with PFS or OS.

## Discussion

To date, there have been few studies on adult patients with unifocal LCH. One contemporary case series studies included 44 patients [8]. This study is one of the largest single center study of unifocal LCH in adults to date. In our series, males were approximately 1.5 times as many as females, and this result was slightly lower than that of overall LCH patients as we have reported before [12]. Regarding the affected organs, the bone, pituitary and lymph nodes were the three most common organs, which was not the same as reported in pediatric patients with LCH. In pediatric patients, the most common affected organs were bone, skin and pituitary [4].

In our previous study, the overall percentage of *BRAF* and *MAP2K1* mutations detected through target gene sequencing of overall adult LCH patients was nearly 90% [7], but it was 59.3% in unifocal LCH patients in adults. Genetic testing was relatively difficult in bone tissue, which may result in the relatively low mutation frequency and VAF values of alterations. In particular, the frequency of *BRAF*^*indel*^ identified in our study was markedly lower than that described in adults (28.8%) [7] and adolescents (50%) [13] with LCH, indicating that *BRAF*^*indel*^ may be uncommon in patients with unifocal LCH in adults. These findings propose that the biology of unifocal LCH may differ from that of MS LCH.

A previous case report found five cases involving liver only, one of them was adult, who presented with biliary tract disease and died from disease progression [14]. Notably, we reported three patients with unifocal liver involvement. Two patients achieved complete response after cytarabine monotherapy. These results indicate that liver involvement can potentially be managed successfully with systemic therapy in patients with unifocal LCH, and may not represent an adverse prognostic factor in this subgroup.

In this study, we described the natural history of unifocal LCH in adults, focusing on the progression of disease in different affected organs. Notably, the rates of progression of pituitary involvement were remarkably high in our series, for reasons that remain unclear. Close monitoring of patients with pituitary LCH for potential systemic progression is warranted. In our study, bone lesions demonstrated a relatively low rate of progression. This is consistent with prior reports indicating that solitary bone LCH lesions often follow an indolent course. Specifically, one study of 61 adults with single-system bone LCH reported only 2 cases of recurrence after complete surgical excision [15]. Another study of 132 pediatric patients found that patients with single-system bone LCH had superior outcomes compared to those with multifocal bone or multi-organ disease [16]. However, a study of 44 adults with unifocal LCH found higher recurrence rates of 25% for initial bone involvement [8]. Studies have reported that the recurrence rate of skin involvement in adults with unifocal LCH was 36% [8], but the skin lesions appeared stable in our patients.

We have previously reported that adolescents with LCH are more prone to disease progression [13], while the 3-year event-free survival of only 54.7% in those with SS-M or MS disease in adults [12]. In this study, we found that patients diagnosed before 30 years of age had significantly shorter PFS. This suggests that young adults may exhibit disease behaviour more similar to children, with more aggressive LCH. We also found that mortality in adults with unifocal LCH was low, consistent with other reports [8, 12].

The primary limitation of this study is that it is a single-centre retrospective study. Additionally, molecular testing was not performed for all patients, limiting the generalizability of these results to the overall unifocal LCH population. However, with a relatively large sample size and lack of prospective studies in this population, our findings provide clinically useful insights. Another limitation was that only 32 patients underwent NGS testing. Additionally, some mutations with variant allele frequencies approaching 50% were not assessed for germline origin.

## Conclusions

In this study, we described the natural history and molecular features of unifocal LCH in adults. The overall percentage of *BRAF* and *MAP2K1* mutations in unifocal LCH among adults was relatively low compared to that reported in the general adult LCH populations. While *BRAF*^*indel*^ were associated with MS disease among adults in our prior study, we did not detect any such association in our unifocal LCH cohort. Age over 30 years at diagnosis was associated with superior prognosis relative to younger patients. The overall prognosis was excellent. Our study suggests that unifocal LCH represents a distinct entity in adults. Future studies should aim to identify high-risk individuals so that targeted surveillance strategies can be employed.

### Supplementary Information

**Additional file 1: Table S1.** 183 candidate genes for target sequencing. **Table S2.** Mutations of adult patients with unifocal LCH.

## Data Availability

Individual participant data will not be available. Study protocol will be available beginning 9 months and ending 36 months following article publication at caoxinxin@pumch.cn .

## Abbreviations

LCH: Langerhans cell histiocytosis
SS-M: Single system multifocal
MS: Multisystem
FDG-PET: Fluorodeoxyglucose positron emission tomography
CT: Computed tomography
MRI: Magnetic resonance imaging
MA: Methotrexate combined with cytarabine
VP: Vindesine and prednisone
PFS: Progression-free survival
OS: Overall survival
ALT: Alanine aminotransferase
AST: Aspartate transaminase
ALP: Alkaline phosphatase
GGT: γ-Glutamyl transpeptidase
