# Supplementary material for: Clinical features, genomic profiling, and outcomes of adult patients with unifocal Langerhans cell histiocytosis
Source: Orphanet J Rare Dis. 2023 Nov 30;18:372. doi: 10.1186/s13023-023-02989-8 (PMC10691033; doi:10.1186/s13023-023-02989-8)
Supplement: Supplementary file 1 — Additional file 1: Table S1. 183 candidate genes for target sequencing. Table S2. Mutations of adult patients with unifocal LCH. [file 13023_2023_2989_MOESM1_ESM.docx]

Additional file 1: Table S1. 183 candidate genes for target sequencing

| ALK | ANKRD50 | ANO10 | AP3B1 | ARAF | ARID1B |
| --- | --- | --- | --- | --- | --- |
| ASXL1 | ATM | ATP4A | ATP9A | ATRX | B2M |
| BCL1 | BCL2L1 | BCOR | BCORL1 | BIRC3 | BOB |
| BRAF | BRD4 | CALR | CASK | CBL | CCDC168 |
| CCR7 | CD274 | CDC73 | CDH1 | CDK6 | CDKN1A |
| CDKN2A | CDKN2B | CEBPA | CEP85 | CHEK2 | CHMP1A |
| COL6A6 | CSF1R | CSF3R | CTNNB1 | DKK2 | DNAH7 |
| DNMT3A | DUSP4 | ECE1 | EFNA4 | EGFR | EMSY |
| EOMES | ERBB2 | ERBB3 | ERBB4 | ETV6 | EZH2 |
| FAS | FBXW7 | FCGBP | FGFR2 | FGFR3 | FLT3 |
| FOXP3 | GATA1 | GATA2 | GNAQ | GNAS | GREB1L |
| GRK4 | HLA-DQA1 | HLA-DQB2 | HNF1A | HRAS | IDH1 |
| IDH2 | ING1 | ITIH6 | JAK1 | JAK2 | KDM5A |
| KDM6A | KDR | KIF5B | KIR2DL4 | KIT | KMT2B |
| KMT2D | KMT2E | KRAS | LYST | MAP2K1 | MAP3K1 |
| MAPK1 | MAPK8 | MAPK14 | MDM2 | MDM4 | MET |
| MLH1 | MSH2 | MSH6 | MUNC13-4 | MYC | MYD88 |
| NCOA2 | NCOA4 | NCOR2 | NEMO | NF1 | NFKB1 |
| NOTCH1 | NOTCH2 | NRAS | NT5DC3 | NTRK1 | PAX5 |
| PBRM1 | PBX1 | PCDHA8 | PDCD1 | PDCD1LG2 | PDCD2 |
| PDGFRA | PHIP | PICK1 | PIK3CA | PIK3CD | PIK3R2 |
| PKNOX2 | PLXNA2 | PMS2 | PNISR | POLD1 | POLE |
| PRF1 | PTEN | PTPN11 | RAB27A | RAF1 | RB1 |
| RBM10 | RET | RICTOR | ROS1 | RPS6 | RUNX1 |
| RYR2 | SCN1A | SEC62 | SETBP1 | SETD2 | SF3B1 |
| SH2D1A | SHOC2 | SLC10A6 | SLC29A3 | SMAD6 | SMPD1 |
| SOS1 | SPRED1 | SREBF1 | SRSF2 | ST8SIA1 | STAG2 |
| STAT3 | STK11 | STX11 | STXBP2 | TAP2 | TERT |
| TET2 | TLR7 | TLR8 | TP53 | TRBV20OR9-2 | TSC1 |
| TTN | U2AF1 | UNC13B | UNC13D | VCL | VEGFA |
| WT1 | XIAP | ZRSR2 |  |  |  |

Additional file 1: Table S2. Mutations of adult patients with unifocal LCH

| **Study ID** | **Gene** | **Transcript** | **Exon** | **Genomic Variant** | **Amino Acid Change** | **VAF** |
| --- | --- | --- | --- | --- | --- | --- |
| unifocal LCH_01 | ERBB3 | NM_001982 | exon3 | c.310G>A | p.V104M | 62.8% |
| unifocal LCH_02 | MAP2K1 | NM_002755 | exon3 | c.302_307del | p.E102_I103del | 8.30% |
| unifocal LCH_03 | PIK3CA | NM_006218 | exon10 | c.1633G>A | p.E545K | 1.0% |
| unifocal LCH_03 | TP53 | NM_000546 | exon6 | c.659A>G | p.Y220C | 5.5% |
| unifocal LCH_04 | BRAF | NM_004333 | exon12 | c.1501G>A | p.E501K | 4.3% |
| unifocal LCH_05 | BRAF | NM_004333 | exon11 | c.1397G>A | p.G466E | 5.6% |
| unifocal LCH_05 | MYC | NM_002467 | exon2 | c.218C>T | p.T73I | 0.75% |
| unifocal LCH_06 | NRAS | NM_002524 | exon3 | c.179G>A | p.G60E | 0.95% |
| unifocal LCH_06 | DNMT3A | NM_022552 | exon19 | c.2311C>T | p.R771X | 0.63% |
| unifocal LCH_06 | KRAS | NM_004985 | exon2 | c.35G>A | p.G12D | 2.9% |
| unifocal LCH_07 | BRAF | NM_004333 | exon15 | c.1799T>A | p.V600E | 0.23% |
| unifocal LCH_07 | PTEN | NM_000314 | exon7 | c.697C>T | p.R233X | 1.20% |
| unifocal LCH_07 | KRAS | NM_004985 | exon3 | c.175G>A | p.A59T | 0.40% |
| unifocal LCH_08 | BRAF | NM_004333 | exon15 | c.1799T>A | p.V600E | 4% |
| unifocal LCH_08 | PTEN | NM_000314 | exon6 | c.517C>T | p.R173C | 2.3% |
| unifocal LCH_09 | PIK3CA | NM_006218 | exon2 | c.323G>A | p.R108H | 18.7% |
| unifocal LCH_09 | NOTCH1 | NM_017617 | exon27 | c.5161G>A | p.V1721M | 11.8% |
| unifocal LCH_10 | ATM | NM_000051 | exon13 | c.1960C>T | p.Q654X | 3.8% |
| unifocal LCH_11 | MAP2K1 | NM_002755 | exon3 | c.302_307del | p.E102_I103del | 8.30% |
| unifocal LCH_11 | ATM | NM_000051 | exon35 | c.5188C>T | p.R1730X | 0.75% |
| unifocal LCH_12 | PIK3CA | NM_006218 | exon10 | c.1633G>A | p.E545K | 0.49% |
| unifocal LCH_13 | CBL | NM_005188 | exon9 | c.1384C>T | p.R462X | 14.1% |
| unifocal LCH_14 | EGFR | NM_005228 | exon4 | c.461C>T | p.P154L | 6.4% |
| unifocal LCH_14 | MAPK8 | NM_001278547 | exon3 | c.132T>A | p.Y44X | 4.2% |
| unifocal LCH_14 | PIK3CD | NM_005026 | exon15 | c.1906C>T | p.R636W | 3.5% |
| unifocal LCH_15 | BRAF | NM_004333 | exon15 | c.1799_1800delTGinsAT | p.V600D | 7.9% |
| unifocal LCH_15 | SLTM | NM_024755 | exon10 | c.1339C>T | p.R447C | 48.8% |
| unifocal LCH_16 | BRAF | NM_004333 | exon15 | c.1799T>A | p.V600E | 5.5% |
| unifocal LCH_17 | BRAF | NM_004333 | exon15 | c.1799T>A | p.V600E | 2.5% |
| unifocal LCH_18 | NRAS | NM_002524 | exon2 | c.37G>A | p.G13S | 2.00% |
| unifocal LCH_18 | NRAS | NM_002524 | exon2 | c.35G>A | p.G12D | 1.10% |
| unifocal LCH_18 | MAP2K1 | NM_002755 | exon3 | c.302_307del | p.E102_I103del | 4.90% |
| unifocal LCH_19 | BRAF | NM_004333 | exon15 | c.1799T>A | p.V600E | 11.3% |
| unifocal LCH_19 | ALK | NM_004304 | exon1 | c.386G>T | p.G129V | 48.6% |
| unifocal LCH_19 | KDR | NM_002253 | exon17 | c.2440G>A | p.D814N | 47.9% |
| unifocal LCH_20 | BRAF | NM_004333 | exon15 | c.1799T>A | p.V600E | 21.3% |
| unifocal LCH_20 | IDH1 | NM_005896 | exon4 | c.297A>G | p.I99M | 48.8% |
| unifocal LCH_21 | MAP2K1 | NM_002755 | exon17 | c.303_308delGGAGAT | p.E102_I103del | 12.5% |
| unifocal LCH_21 | PHIP | NM_017934 | exon5 | c.229C>T | p.Q77* | 7.8% |
| unifocal LCH_21 | TET2 | NM_017628 | exon3E | c.3451G>T | p.E1151* | 46.3% |
| unifocal LCH_21 | TP53 | NM_001126118 | exon9 | c.886C>G | p.R296G | 5.8% |
| unifocal LCH_22 | MAP2K1 | NM_002755 | exon3 | c.303_308delGGAGAT | p.E102_I103del | 4.6% |
| unifocal LCH_22 | ERBB4 | NM_005235 | exon17 | c.1972T>A | p.I658F | 49.5% |
| unifocal LCH_22 | MAPK8 | NM_139046 | exon4 | c.110A>C | p.Q37P | 6.3% |
| unifocal LCH_23 | BRAF | NM_004333 | exon15 | c.1799T>A | p.V600E | 6.7% |
| unifocal LCH_24 | TP53 | NM_001126118 | exon6 | c.617G>A | p.G206D | 0.53% |
| unifocal LCH_24 | BRAF | NM_004333 | exon15 | c.1796C>T | p.T599I | 0.3% |
| unifocal LCH_24 | CBL | NM_005188 | exon1 | c.173A>G | p.K58R | 46.6% |
| unifocal LCH_25 | BRAF | NM_004333 | exon15 | c.1799T>A | p.V600E | 2.9% |
| unifocal LCH_25 | ALK | NM_004304 | exon15 | c.2584G>C | p.E862Q | 47.5% |
| unifocal LCH_25 | NOTCH1 | NM_017617 | exon26 | c.4732_4734del | p.V1578del | 2.2% |
| unifocal LCH_26 | MAP2K1 | NM_002755 | exon2 | c.120_161delAGAGCTTGATGAGCAGCAGCGAAAGCGCCTTGAGGCCTTTCT | p.E41_L54del | 2.4% |
| unifocal LCH_26 | ALK | NM_004304 | exon1 | c.511A>C | p.S171R | 4.1% |
| unifocal LCH_26 | ERBB4 | NM_005235 | exon12 | c.1487G>A | p.C496Y | 3.2% |
| unifocal LCH_26 | NOTCH2 | NM_024408 | exon21 | c.3474C>A | p.C1158* | 4.9% |
| unifocal LCH_26 | PMS2 | NM_000535 | exon11 | c.921dupA | p.D308Rfs*44 | 2.6% |
| unifocal LCH_27 | BRAF | NM_004333 | exon15 | c.1799T>A | p.V600E | NA# |

# This sample was measured by fluorescence quantitative PCR
